# Sinus of Valsalva Aneurysm Rupture: An Unusual Presentation of Chromosome 22q11.2 Deletion: A Case Report

**DOI:** 10.1155/2012/387075

**Published:** 2012-09-23

**Authors:** Eda-Cristina Abuchaibe, Nancy Dobrolet, Katherine Peicher, Roque Ventura, Elizabeth Welch

**Affiliations:** ^1^Department of Pediatric Medical Education, Miami Children's Hospital, 3100 SW 62nd Avenue, Miami, FL 33155-3009, USA; ^2^Department of Pediatric Cardiology, Miami Children's Hospital, 3100 SW 62nd Avenue, Miami, FL 33155-3009, USA; ^3^Department of Osteopathic Medicine, Nova Southeastern University, Davie, FL 3314-7796, USA

## Abstract

Sinus of Valsalva aneurysm (SVA) is defined as a weakness in the aortic valve wall, immediately above the attachments of each of the aortic cusps. This weakness can rupture and create an aortocardiac fistula. There are many congenital heart defects associated with chromosome 22q11 deletion, especially involving the aortic arch and its branches. SVA is not an anomaly usually associated with chromosome 22 deletion. We report the case of a 19-year-old female who presented to our institution with SVA rupture. She was subsequently diagnosed with chromosome 22q11 deletion syndrome. Despite dysmorphic facial features and a learning disability, our patient had not been diagnosed with the chromosome abnormality. SVA is a rare congenital heart defect and has only once previously been reported in a child with a chromosome 22q11 deletion. We report the first case where aneurysm rupture preceded the chromosomal findings. Chromosome 22q11 deletion could be missed due to either the unfamiliarity of physicians with the syndrome or the variability and subtlety of the phenotype. This was demonstrated by our patient who, at age 19 after presenting with an SVA rupture, prompted physicians to find an explanation for her coexisting dysmorphic features and her learning disability.

## 1. Introduction

22q11.2 deletion syndrome (DiGeorge, conotruncal facial, Shprintzen, and Velocardiofacial syndrome) is a common syndrome that is characterized by deletion of the chromosome 22, in the location designated q11.2 [1]. There are many congenital heart defects ranging from mild to severe, which are associated with chromosome 22q11.2 deletion. Sinus of Valsalva aneurysm (SVA) has not been associated with the syndrome.

**Figure 1:**
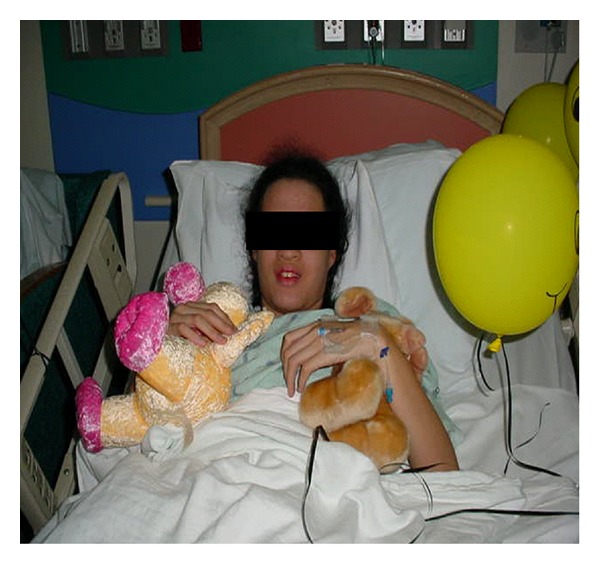
Dysmorphic facial features: high prominent forehead, long nose with a broad bridge, mild micrognathia, and short palpebral fissures.

We present a case of a SVA rupture, in a patient who was noted to have dysmorphic facial features in addition to a history of learning disability, which subsequently lead to a diagnosis of chromosome 22q11.2 deletion syndrome (Figure 1). We present this case to illustrate the importance of recognizing patients with variable dysmorphic features associated with this chromosome deletion and the importance for lifelong cardiology followup. Identification of the chromosome deletion is now readily available by Fluorescence in situ hybridization (FISH) studies. 

## 2. Case Report

**Figure 2:**
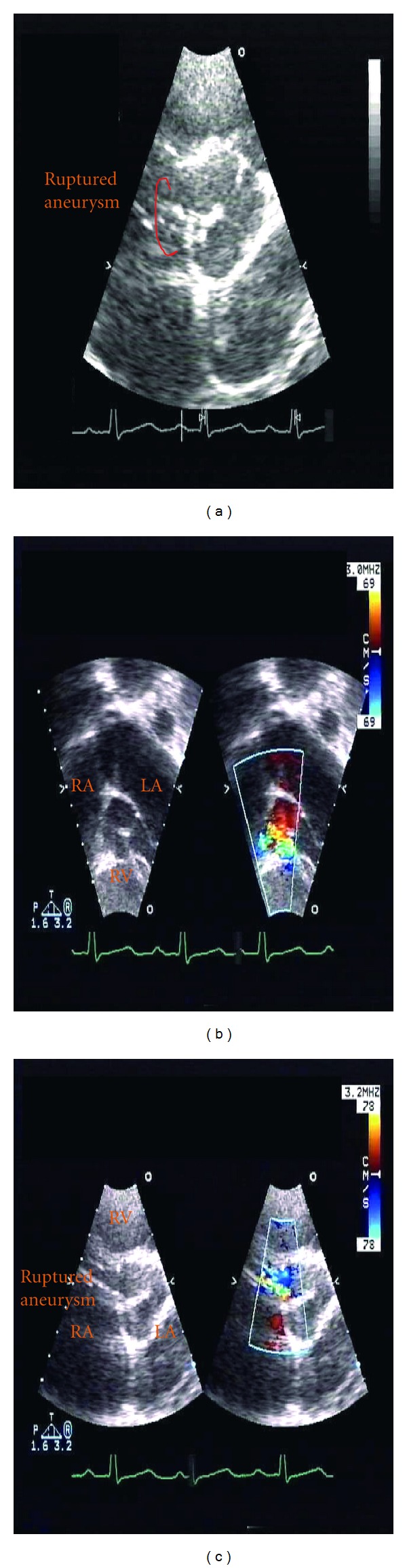
(a) short-axis echo view. Demonstrating fingerlike protrusion evident of ruptured right coronary sinus of Valsalva aneurysm. (b) Subcostal view. (c) Short-axis view of color compared with color flow doppler evidence of aorta to right ventricle fistulous connection.

**Figure 3:**
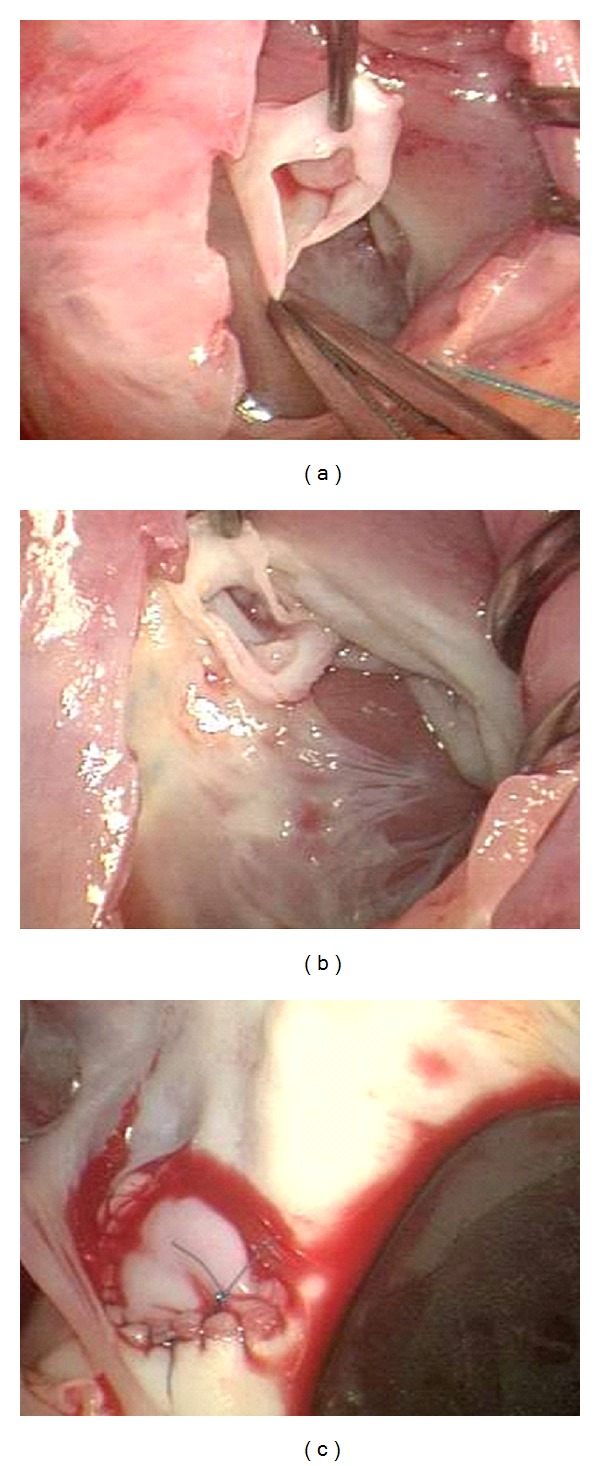
(a) Intraoperative image demonstrating ruptured right sinus aneurysm with visualization of the intact noncoronary cusps. (b) Intraoperative picture demonstrating the 1 cm hole at the junction of the right coronary cusp and at the junction of the right noncoronary commissures. (c) Intraoperative picture after closure of the rupture with bovine pericardium patch.

A nineteen-year-old previously healthy female presented to an emergency department (ED) with a two-week history of shortness of breath, especially when climbing stairs. Three days prior to presentation, she complained of palpitations. Her past medical history was significant only for a learning disability. On physical exam, the patient was noted to be anxious, with mild pallor. BP 117/55 HR 119, O_2_ sat 100% in room air. Cardiovascular examination was unremarkable except for her tachycardia. Her electrocardiogram demonstrated sinus tachycardia with a right ventricular conduction delay. On day 3 of admission, a 3/6 continuous murmur was noted along the right sternal border with extension to the axilla. P2 was also prominent. A transthoracic echocardiogram (TTE) and transesophageal echocardiogram (TTE) demonstrated a fistulous connection between the aorta and right ventricle (Figure 2). Uepon arrival to the Cardiac Intensive Care Unit, the patient's symptoms progressed and she became uncomfortable in the supine position secondary to SOB. Intensive care monitoring revealed a widened pulse pressure. The above described murmur was also heard on physical exam, we also noticed that she displayed dysmorphic features including a highly placed and prominent forehead, long nose with broad bridge, mild micrognathia, high–arched palate and short palpebral fissures. The patient also had significant delay in motor and cognitive milestones. Because of the above physical and developmental findings, chromosome analysis, FISH and amino acid analysis for homocystinuria were added to the admission labs. A transthoracic echocardiogram (TTE) was performed confirming a SOV rupture to the right ventricle with possible extension to include the right atrium and moderate aortic insufficiency. At the time of surgery, 1 cm hole was noted at the junction of the right coronary and non-coronary commissures with communication to the right ventricle and atrium. The repair consisted of a bovine patch closure of this hole, excision of the aneurysm and closure of the aorta with multiple layers of prolene (Figure 3). A post-operative TEE revealed complete closure of the aneurysm. There was no significant residual aortic insufficiency, and good biventricular systolic function. Postoperative complications included moderate hypertension, controlled with an ace inhibitor, transient hypocalcemia and a fungal rash over 1/4 of sternotomy site necessitating treatment with oral antifungal medication. Chromosome 22q11.2 microdeletion was identified by the FISH test, prior to discharge. The patient continues to be followed by cardiology, endocrinology and genetics. 

## 3. Discussion 

SVA was first described by John Thurman in 1840 [2]. The incidence is rare, occurring in approximately 0.1% to 3.5% of the general population [3], with a male to female ratio of 4 : 1 ratio. The defect has been associated with a congenital deficiency in elastin tissue and abnormal bulbus cordis [4]. This deficiency leads to a separation between the aortic media and annulus fibrous. Dilation of the aorta with the formation of a diverticulum may occur in one or more sinuses of Valsalva [2]. 95% of all SVA originate along the right or noncoronary sinus [2]. SVAs are usually asymptomatic if not ruptured, though there have been reports of symptoms associated with very large unruptured aneurysm such as right ventricular outflow tract obstruction and complete heart block [3]. Rupture of SVA has been reported in patients between 11 and 67 years of age with a mean age of 35.8 years [5]. A small rupture is associated with progressive shortness of breath (SOB) or mild congestive heart failure. Extension of the rupture or a large ruptured sinus can lead to a major cardiovascular event that demands prompt diagnosis and treatment [2]. Given the relative infrequency of this defect achieving a definitive diagnosis can be quite challenging [5]. Defects of progressive aortic dilation have a higher incidence of SVA with and with out rupture. SVAs have been reported in heart lesions such as Tetralogy of Fallot, transposition of the great arteries (postarterial switch procedure), hypoplastic left heart syndrome (postpalliation), subpulmonary ventricular septal defects, and bicuspid aortic valve [6]. Syndromes such as Marfans, Loeys-Dietz, Ehlers-Danlos [4], Turners, Noonan's [7], homocystinuria, and most recently chromosome 22 q11.2 microdeletion syndrome [8] are known to have progressive aortic root and ascending aorta dilatation. SVAs with rupture have also been reported in the above syndromes. 

Chromosome 22q11.2 microdeletion is a common chromosome deletion affecting approximately 1/4000 live births [6]. It was described by Dr. Angelo DiGeorge in the mid 1960s who described infants with cardiac defects, abnormal facies, cleft palate, velopharyngeal incompetence, thymic hypoplasia or aplasia, associated T-cell deficiency, and hypoparathyroidism leading to low calcium levels [9]. Developmental delay, mental retardation, and psychiatric disorders are features that are identified as the child grows [9]. The genetic changes associated with 22q11.2 are still under investigation, but researchers have so far determined that the loss of *TBX1* gene on chromosome 22 is probably responsible for many of the characteristics of the syndrome [10]. Cardiac defects present in 80% of patients [6] with most cases of the syndrome presenting in the newborn period. FISH studies are becoming part of the standardized diagnostic workup especially in cardiac intensive care units especially in infants who present with conotruncal cardiac anomalies such as Tetralogy of Fallot, truncus arteriosus, interrupted aortic arch B, misaligned ventricular, and subpulmonary ventricular defects. Most recently, a wide range of complex aortic arch anomalies, have been reported in patients with the chromosome 22q11.2 microdeletion syndrome [8]. It is certainly possible that patients who lack the usual congenital heart disease associated with the chromosome deletion are missed and lost to followup. Mc Elhinney et al. reported as many as 38% of patients with chromosome 22.q11 deletion syndrome are diagnosed past the age of 6 months [11]. Although sinus of valsalva aneurysm has not been linked to a specific chromosome, chromosome 22q11.2 microdeletion syndrome has been linked to progressive ascending aortic dilatation [8]. This is the second reported case of ruptured SVA in chromosome 22q11.2 syndrome, the first case was reported in the Spanish literature in a 21-year-old patient with known chromosome 22q11.2 microdeletion syndrome. However, our patient is the first report in the American literature and differs in that the diagnosis of the chromosome microdeletion was made in late adolescence and at the time of her hospitalization. Diagnosis was suspected based on her phenotypical findings. 

In conclusion, we propose that all patients with chromosome 22q1.2 deletion syndrome, with or without heart disease, receive mandatory and lifetime cardiology followup. The relative high frequency of progressive aortic dilatation in these patients can lead to other complications such as SVA or other acquired aortopathies [8]. Early identification of chromosome 22q11.2 microdeletion allows for early detection of both cardiac- and noncardiac- associated problems as well as appropriate genetic counseling for the patient and their family, with lifelong cardiology followup despite the lack of significant heart disease at birth.

## Abbreviations

SOVA: Sinus of Valsalva aneurysm
ED: Emergency department
TTE: Transthoracic echocardiogram
TEE: Transesophageal echocardiogram
MCH: Miami Children's Hospital
OR: Operating room
SEM: Systolic ejection murmur
CICU: Cardiac intensive care unit.
